# Application of intraoperative ultrasound in the resection of high-grade gliomas

**DOI:** 10.3389/fneur.2023.1240150

**Published:** 2023-10-26

**Authors:** RenJie Wei, Hao Chen, YuXiang Cai, JingCao Chen

**Affiliations:** ^1^Department of Neurosurgery, Zhongnan Hospital of Wuhan University, Wuhan, China; ^2^Department of Pathology, Zhongnan Hospital of Wuhan University, Wuhan, China

**Keywords:** review, intraoperative ultrasonography (IOUS), contrast-enhanced ultrasound (CEUS), high-grade glioma (HGG), surgical outcome

## Abstract

The incidence of gliomas is approximately 3–5/100,000, with high-grade gliomas accounting for approximately 30–40% of these tumors. Surgery is a confirmed positive factor in prolonging the survival of these patients, and a larger resection range means a longer survival time. Therefore, surgery for high-grade glioma patients should aim to maximize the extent of resection while preserving neurological function to achieve a better quality of life. There is consensus regarding the need to lengthen progression-free survival (PFS) and overall survival (OS) times. In glioma surgery, methods such as intraoperative computed tomography (ICT), intraoperative magnetic resonance imaging (IMRI), navigation, 5-aminolevulinic acid (5-ALA), and intraoperative ultrasound (IOUS) are used to achieve an expanded resection during the surgical procedure. IOUS has been increasingly used in the surgery of high-grade gliomas and various tumors due to its convenient intraoperative use, its flexible repeatability, and the relatively low cost of operating room construction. With the continuous upgrading of ultrasound equipment, IOUS has been able to better assist surgeons in achieving an increased extent of resection. This review aims to summarize the application of ultrasound in the surgery of high-grade gliomas in the past decade, its improvement in patient prognosis, and its prospects.

## Introduction

Glioma is the most common primary brain tumor in the adult intracranial region, with an incidence of approximately 3–5/100,000. It can occur at any age and at any location in the central nervous system, but it affects mainly the intracranial region in individuals aged 50–60 years (1–4). Intracranial gliomas can be classified into different types based on the source cells (astrocytes or oligodendrocytes), and according to the WHO histological classification, they are divided into grades 1–4. Among them, grades 3 and 4 are categorized as high-grade gliomas (HGGs) (4).

Despite the rapid development of surgical techniques and basic medical research, high-grade gliomas such as glioblastomas still have high morbidity and mortality rates, with a 5-year survival rate of <5% (5). Gross total resection (GTR) of gliomas is a clear and important factor in prolonging progression-free survival (PFS) and overall survival (OS); thus, the pursuit of GTR is an appropriate goal for the surgeon. However, depending on the location of the glioma, GTR becomes unrealistic when considering intra-tumoral or peritumoral tissues, such as functional areas and blood vessels.

Poor outcomes are seen in both nodular and diffuse growth patterns in HGGs. With tools such as preoperative and intraoperative MRI, neuro-navigation with imaging, and intraoperative fluorescence, surgeons have achieved an increased extent of resection, and PFS and OS are prolonged (3, 6–8).

Ultrasound has been widely used in abdominal surgery and gynecology, with a history of mature application that predates its use in neurosurgery. Reports of the use of ultrasound in neurosurgery can be traced back to the 1980s or even earlier (9). The brain itself is soft and elastic, which allows good contact with the ultrasound probe. However, due to the limited imaging quality of the equipment available at the time, mainly manifesting as low resolution and artifacts, intraoperative ultrasound (IOUS) did not gain widespread use in neurosurgery. In the past decade, with updates in equipment, IOUS has been increasingly used in neurosurgical procedures (10).

A quick review of the principles, classification, and characteristics of ultrasound in neurosurgery is carried out as follows. Ultrasound has been widely used in the examination of abdominal organs such as the liver, gallbladder, and pancreas, as well as in gynecological examinations. It is also effective in detecting lesions in the thyroid tissue. Ultrasound is a non-invasive imaging technique that utilizes high-frequency sound waves with short wavelengths to produce images of internal bodily structures. These sound waves, also known as ultrasound waves, have frequencies mostly between 2 and 15 MHz and are beyond the range of human hearing. As they pass through different tissues in the body, they lose varying amounts of energy, which is reflected back to the probe and captured. The transducer then converts these sound waves into electric and video signals that are processed and displayed on a screen as images.

The short ultrasound wavelength allows the waves to exhibit good anisotropy, meaning they can penetrate opaque substances, such as the human body. As they pass through tissues, they undergo reflection and refraction, which may affect the quality of the image produced.

Additionally, the absorption of ultrasound waves by human tissues varies depending on their shape and properties, resulting in attenuation of the sound waves as they travel through the body.

Ultrasound diagnosis relies on various imaging methods, with B-mode and D-mode being the most commonly used techniques. B-mode ultrasound involves converting reflection signals from the tissues of the body into dots of varying brightness and displaying them as a two-dimensional image on a flat screen. Due to its excellent intuitiveness and ease of replication, B-mode ultrasound has become widely used in medical diagnostics. Moreover, by using a pre-calibrated 2D phased array probe, it is possible to generate three-dimensional (3D) images based on B-mode ultrasound. This imaging technique involves acquiring 200–300 images while tilting the probe, which are then reconstructed to create a 3D volume that provides a more intuitive representation of tumors and surrounding structures (11).

The use of 3D ultrasound imaging has several advantages over traditional 2D imaging techniques. For instance, 3D ultrasound allows for a better visualization of the tumor's location and size, as well as its relationship with surrounding structures. Additionally, 3D ultrasound can facilitate easier tumor resection planning and improve the accuracy of surgical procedures. Despite the numerous benefits of 3D ultrasound, there are also some limitations to this technique. One significant limitation is the need for specialized equipment and trained personnel to perform and interpret the 3D images accurately. Additionally, the quality of the 3D image may be influenced by factors such as the angle and position of the probe, as well as the acoustic properties of the surrounding tissues (12, 13).

Doppler ultrasound, also known as D-mode, is a sensitive imaging technique that is particularly useful in visualizing blood vessels and measuring the direction and flow of blood. This is because Doppler ultrasound can detect the motion of blood flow, allowing for accurate measurement of blood flow velocity and direction. Recent advancements in algorithms and instrumentation have led to the development of advanced Doppler ultrasound technology that enables physicians to visualize and characterize tumor microvasculature, which is significant because the growth and spread of tumors are heavily reliant on the formation of new blood vessels or angiogenesis. Moreover, the use of advanced Doppler ultrasound technology has several advantages over traditional Doppler ultrasound techniques. For instance, advanced Doppler ultrasound technology allows for the visualization of smaller blood vessels with higher resolution, enabling more precise characterization of microvascular structures within the tumor. Additionally, advanced Doppler ultrasound can provide information on blood flow in real time, allowing physicians to monitor changes in tumor perfusion during treatment and adjust therapy accordingly (14, 15).

**Figure 1:**
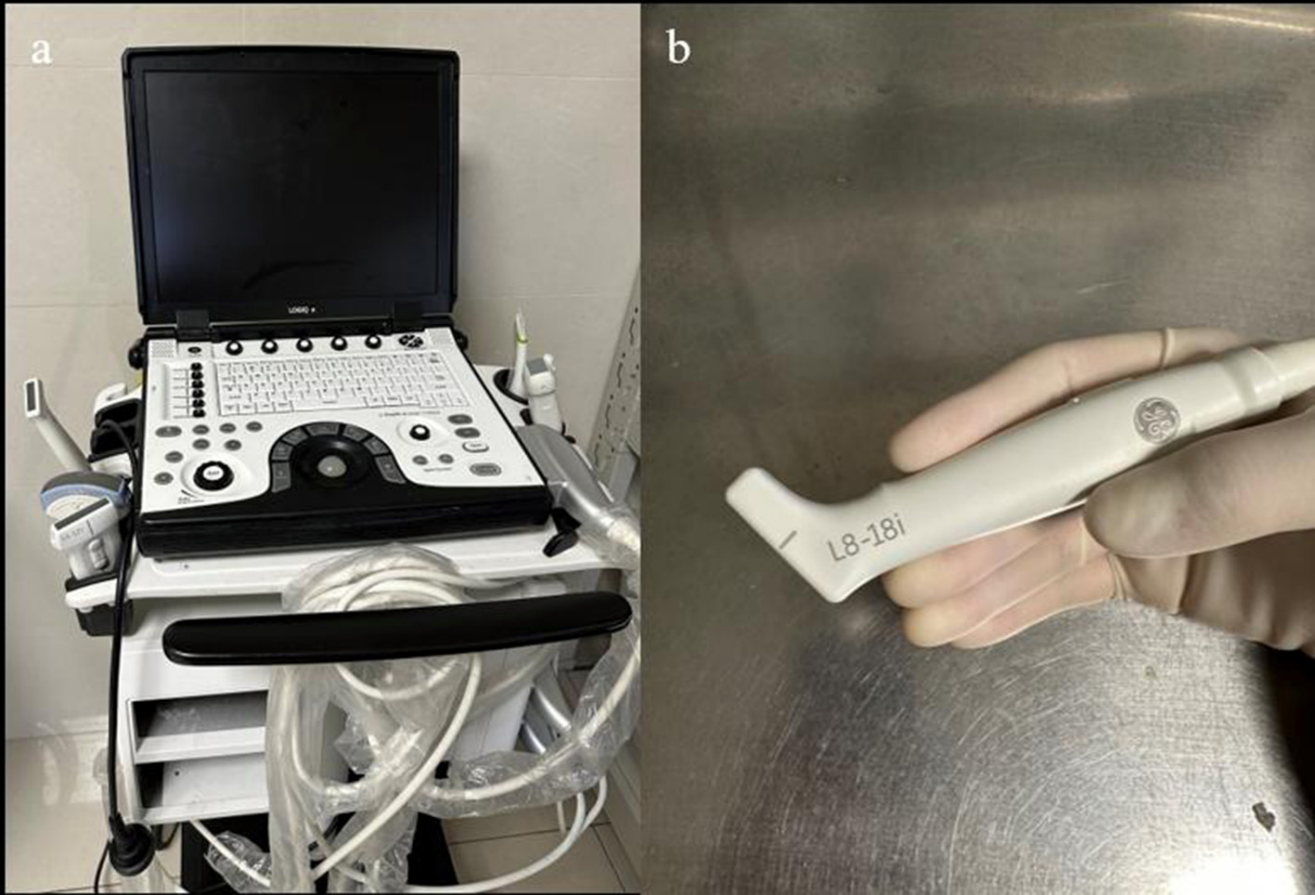
**(a)** Portable ultrasound machine LogicQ (GE, USA) for the IOUS procedure. **(b)** “Hockey bar” linear probe, providing a scan depth of 4.5 mm.

The frequency range of ultrasound probes used in neurosurgery varies depending on the location and depth of the lesion being imaged. For lesions 2–3 cm away from the cortex, a higher frequency range of 10–12 MHz is typically used, while deeper lesions are visualized using a lower frequency range of 5–7 MHz. This variation in frequency allows for optimal imaging of the different depths of the brain tissue. In addition to the frequency range, the type of ultrasound probe used also plays an important role in neurosurgery. High-frequency probes, for instance, are best suited for imaging superficial tumors as they provide high-resolution images with excellent detail. Linear or convex multi-frequency probes, on the other hand, are better suited for deeper lesions as they can penetrate more deeply into the brain tissue (Figure 1). Microconvex or ball-shaped probes are often used for exploring the surgical cavity after tumor resection as they offer a wide field of view with enhanced visualization of nearby anatomical structures. Specialized probes, such as endoscopic ultrasound probes, are also used for procedures involving the pituitary gland as they provide a minimally invasive approach with exceptional visualization of the gland (16). For tumor resection, exposing the surface of the tumor and using a probe to make contact can help avoid damage to blood vessels (12).

Images from the IOUS can be reconstructed into the 3D form, presenting the tissue features in three dimensions. 3D images can be combined with preoperative MRI to form ultrasound navigation. In recent years, more research studies have been done to study the registration of MR images of brain tumors as well as IOUS images (17, 18).

**Figure 2:**
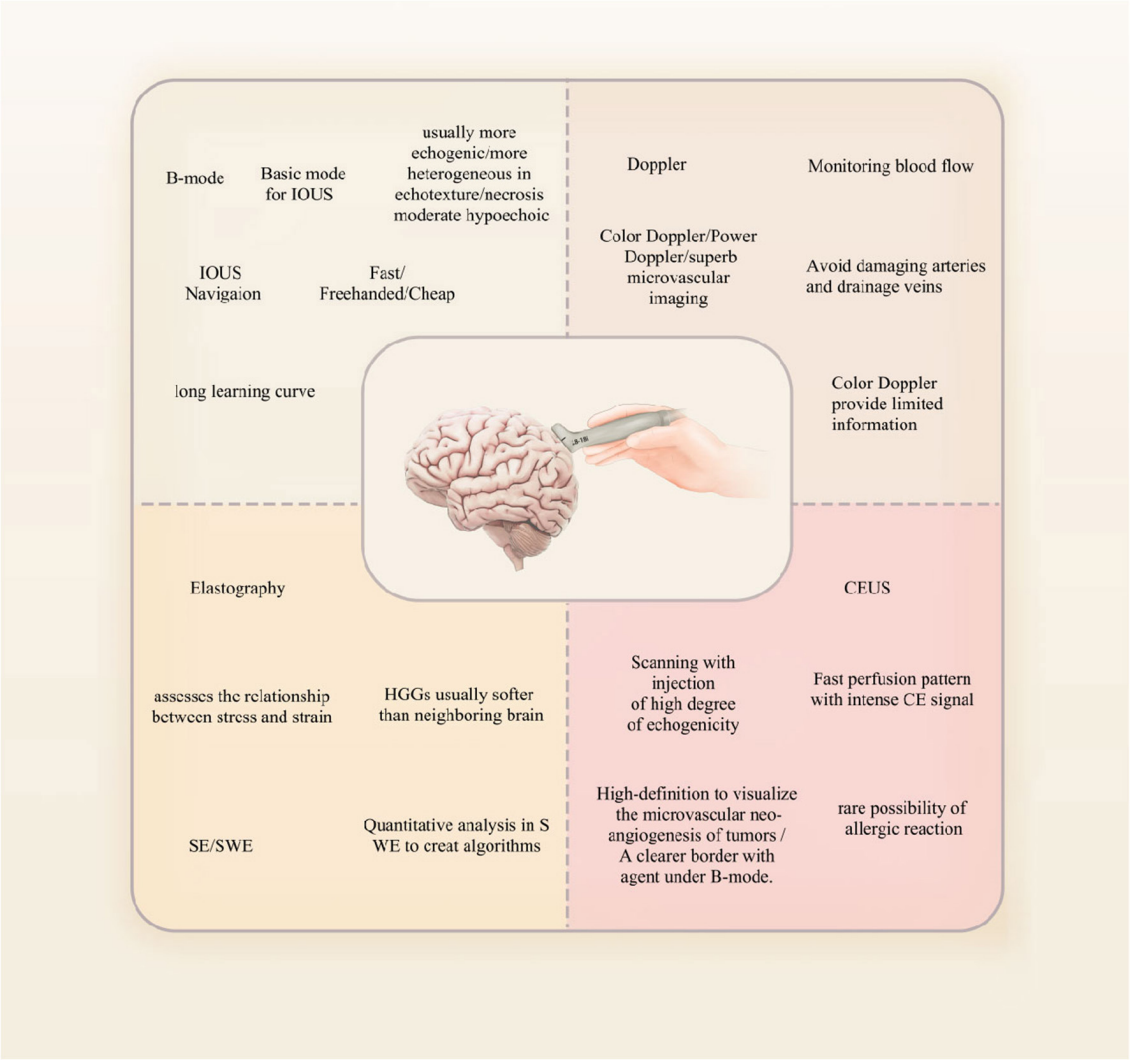
A brief classification of four types of IOUS.

For neurosurgery, contrast-enhanced ultrasound (CEUS) is a relatively new technique that was developed in the last decade and that uses microbubbles or other contrast agents to improve image quality. Because of the difference in the contrast signals between ultrasound and contrast agents and the tissue, contrast agents can be clearly observed. The most clinically used agent is microbubbles, which have a perfluorocarbon gas core and a phospholipid or protein shell. Microbubbles have a diameter ranging from 1 to 10 μm, so they cannot extravasate outside the vasculature. Microbubbles may augment the acoustic impedance between lesions and surrounding tissues by producing highly efficient scattering of waves (19, 20). CEUS allows for more detailed visualization of blood flow and tissue perfusion, particularly in areas where traditional ultrasound imaging may be limited. It is commonly used in the diagnosis and monitoring of liver and kidney diseases as well as in the evaluation of tumors (21–23) (Figure 2).

## IOUS characteristics according to high-grade glioma subtype

Gliomas with WHO grades of 3 and 4 are conventionally considered high-grade gliomas, and they include subtypes such as glioblastoma (GBM), astrocytoma, oligodendroglioma, and diffuse hemispheric glioma. These subtypes differ in histology, cytological features, genetic characteristics, and other aspects (4). High-grade tumors such as grade III-IV gliomas have distinct imaging features on ultrasound. These tumors typically present with heterogeneous internal echoes, possible presence of fluid necrosis areas, irregular shape, indistinct borders, and obvious edematous areas. However, the overall solid mass of high-grade gliomas is usually hyperechoic.

Recent studies by Prada et al. (24) suggested that high-grade gliomas manifest mainly as hyperechoic borders and ISO/hypoechoic central parts. This finding challenges the traditional understanding of the imaging features of high-grade gliomas on ultrasound and highlights the importance of continued research in this area (25–27). Peritumoral edema, which is the swelling around a brain tumor, can sometimes be mistaken for residual tumor tissue on B-mode ultrasound imaging. This can lead to unnecessary brain damage during surgery. Previous studies have suggested that traditional B-mode ultrasound has only limited ability to provide a clear description of the tumor, tumor border, surrounding tissue, edematous tissue, and even residual tumor caused by resection. Artifacts can also be a problem in B-mode ultrasound imaging (6, 28). Grayscale B-mode imaging is particularly useful for recognizing and distinguishing low-grade gliomas (LGGs) from surrounding tissues. LGGs typically have less peritumoral edema, and the boundary between the tumor and adjacent normal brain tissue is not obscured by the edematous tissue. This makes it easier to identify and differentiate LGGs using ultrasound imaging (21, 29, 30). Using B-mode IOUS alone for evaluating the resection extent of HGG during surgery can be challenging since both malignant tumor tissue and peritumoral edema can appear as areas of high echogenicity on ultrasound imaging. While IOUS has been shown to improve outcomes in HGG resection compared to surgery without intraoperative guidance, many researchers consider it inferior to IMRI.

The effectiveness of IOUS tends to decrease during surgery. In contrast, IMRI offers precise resection and a better assessment of the residual tumor tissue. IMRI also provides more accurate information about the location and extent of peritumoral edema, which can help guide the surgical approach and minimize damage to the healthy brain tissue (23, 29–31). In 2013, Mair conducted a preliminary classification of ultrasound images for various tumors based on ontological visibility and boundary visibility. The study included 105 patients, and the ultrasound images of high-grade gliomas were generally classified as grade 2 or higher, indicating good visualization of the solid tumor and varying visualization of the borders. In contrast, the median ontological visibility of low-grade astrocytomas and oligodendrogliomas was grade 2 or lower, suggesting the lower visualization quality of these tumors on ultrasound imaging (32–34). The conclusion of Mair's study suggests that the boundary and ontological imaging effects of LGGs are poorer on ultrasound imaging than those of high-grade gliomas. However, the researcher also acknowledges the study's small sample size. Therefore, further research is needed to validate and refine Mair's classification system for ultrasound imaging of brain tumors, especially for HGGs.

CEUS is a new technology that has emerged in the last decade and is increasingly being used in neurosurgery. Currently, Prada et al. are known to be at the forefront of its application in this field (35). The ultrasound probe captures signals to generate images, and the microbubbles in the contrast agent produce echoes when scanned by ultrasound waves, thereby enhancing local ultrasound signals. The gas inside the bubbles has different acoustic impedances (i.e., density and speed of sound) and forms an interface that reflects back and forth with surrounding tissues. Therefore, strong acoustic reflections and multiple echoes appear during the ultrasound scanning process, enhancing visualization of the area. In addition, ultrasound contrast agents can also enhance ultrasound signals through hemodynamic effects. They circulate through the blood flow to reach specific areas and remain in the blood vessels. When the ultrasound probe scans the area, the vibration of the bubbles causes slight oscillations in the surrounding blood flow, producing stronger ultrasound signals. LGGs typically exhibit mild and punctate enhancement on contrast-enhanced imaging, with a diffuse and scattered appearance and slow or delayed arterial and venous phase responses. HGGS, on the other hand, demonstrates highly enhanced and unevenly distributed multilobulated nodules and rapid perfusion patterns. Due to the different types of blood supply between tumors and the normal brain tissue, CEUS can more accurately locate and depict the margins of gliomas than traditional ultrasound and can also differentiate tumor vasculature. The tumor margins displayed by CEUS are usually larger than those displayed by conventional ultrasound (23).

In the Prada et al. (36) publication comparing CEUS to IMRI for surgical guidance, all GBM lesions demonstrated strong enhancement compared to the normal brain tissue, allowing for clear differentiation between the surrounding brain parenchyma and the tumor. The contrast enhancement kinetics are similar for all GBM lesions, showing rapid arterial perfusion and rapid venous drainage. During the arterial phase (2–3 s), the chaotic transfer of microbubbles within the lesion is observed, and peak enhancement is seen at 5 s. CEUS has a very fast transfer time, with the venous phase occurring at 15 s in all cases. The time to peak and time-to-peak enhancement of high-grade gliomas are earlier than those of edema and the normal brain tissue, making it helpful to differentiate gliomas, peritumoral edema, and the normal brain tissue (37).

Tumors can also be differentiated based on their tissue characteristics using elastography to assess tissue mechanical properties. In strain elastography (SE), mechanical force is applied to measure the rigidity of the lesion, which is then qualitatively evaluated using a color map. In contrast, shear wave elastography (SWE) provides quantitative assessment. This technique uses ultrasound stimulation to induce tissue displacement, providing a quantitative representation of rigidity (38). IOUS is affected by non-linearities in tissue imaging. Non-linearity in ultrasound refers to the effect of material non-linearity on sound waves propagating inside an object during ultrasound imaging. This changes the frequency distribution of the sound waves and affects image quality. Non-rigid registration is a medical image processing technique that matches one or more patients' medical image data with real-time image data of a target patient, thereby applying existing knowledge to new cases. Since the shape, position, and size of tissues and organs vary greatly between different patients, non-rigid deformation and morphological differences must be considered to obtain more accurate registration results (37, 39).

In a single-center study by Cepeda et al. (40), 40 HGG cases were included, and there was a significant difference (*P* < 0.001) in the tumor mean tissue elasticity values between the pathological groups. The main technical limitations found in some study series include artifacts after opening the dura mater, variability in the frequency and amplitude of mechanical pulsation, and uncertainty in evaluating deep lesions (38). According to the latest study by Hou et al. (41), combining the SWE mode with superb microvascular imaging (SMI), it was found that in the SWE mode, HGG and LGG showed completely different values under Young's modulus, with a diagnostic threshold for distinguishing HGG and LGG at 13.05 kPa. In the SMI mode, the tissue surrounding the HGG was described as having distorted blood flow signals, while the HGG tissue itself exhibited dilated and bent vessels. Therefore, SWE and SMI can assist in the diagnosis of tumors before surgery and “early” tumor grading, which can have a positive impact on later treatment (42). According to Del Bene et al. (27) and recent studies, in the SE mode, HGG appears softer than the normal brain tissue, while LGG appears harder than the normal brain tissue (43). As a relatively new technology, some studies have pointed out that SMI can distinguish the healthy brain tissue (characterized by vertically penetrating, fine, straight vessels) from glioblastoma (exhibiting rounding, dilating, and bending vessels), as well as low-grade glioma (displaying fine and straight vessels) (44).

## Surgical outcomes of high-grade glioma patients according to the IOUS application type

IOUS is a valuable tool that can provide real-time visual images to assist in HGG surgery. It enables surgeons to determine the location, size, shape, and other features of the tumor, guide the surgical scope and depth of resection, and assess the residual after surgery. However, due to the similarity in presentation of the different subtypes of HGGs, there is no clear distinction of features in terms of IOUS imaging. The use of ultrasound for high-grade gliomas has been studied to some extent, but researchers have focused more on the outcomes of resection for low-grade tumors and have more conclusively demonstrated that the outcome of resection for low-grade tumors is more favorable (29). For LGG, the 10-year survival rate is 91% when the extent of resection is >90% (45). However, due to the diffuse and irregular growth patterns of high-grade gliomas and the similarity in echoes to peritumoral edema, intraoperative B-mode ultrasound has generally shown poor results compared to IMRI. Munkvold et al. and Solheim et al. believe that both IOUS and IMRI have low sensitivity (Sn) in detecting small residual tumor volumes during surgery for high-grade gliomas (46, 47). In the application of HGG surgery, increasing attention has been given to 3D navigation, image fusion, and other techniques to improve the accuracy and effectiveness of intraoperative imaging. These techniques can help clinicians more accurately visualize and locate tumors, guide the surgical approach, and evaluate the extent of resection.

Recent studies have gradually changed the existing understanding and suggest that B-mode ultrasound can make effective contributions to the complete resection of high-grade gliomas. In a study by Hervey-Jumper and Berger the residual tumor volume under B-mode ultrasound was smaller than that under traditional neuro-navigation methods, and OS and symptom-free survival were comparable to those of traditional navigation surgery (48). Inspired by AI-Holou et al., in a single-center study by Giussani et al., combining new surgical techniques with IOUS-guided resection of GBM surrounding lesions, the GTR rate reached 85% among 40 patients, and the average residual tumor volume after surgery was 1.44 cm^3^ (49, 50). In a 10-year cohort study by Shetty et al. (51), 210 patients who underwent IOUS-guided surgery were studied. Under IGS navigation, there was no significant difference in GTR rates between HGG and LGG. For validation resection rates using ultrasound and MRI, there was also no significant difference between the two (78%/83%). In a literature review, for HGG, IOUS-guided resection combined with navigation technology achieved a GTR rate of 73.6%, while high-field MRI achieved a rate of 68.3% (52). Regarding improving patient survival, some studies have shown that when comparing the effectiveness of techniques such as 5-ALA and IOUS, patients who undergo GTR surgery for GBM using IOUS have a 3-month longer median survival period than those who do not use it.

Similarly, there is an extended effect on PFS (53). Another literature review suggests that the use of IOUS can improve 1- and 2-year survival rates (26, 54). Therefore, ultrasound-guided surgery can perform effective resection of high-grade gliomas more safely and improve patients' OS and PFS. In comparison to IMRI, Coburger's study showed that using a linear array ultrasound probe for assistance during GBM resection had a sensitivity (Sn) that was 20% higher than that of IMRI, but the specificity (Sp) was not significantly different from that of IMRI. For recurrent gliomas, the Sn and Sp of all three detection methods decrease, but there is still no significant difference between linear array IOUS and IMRI (55).

**Figure 3:**
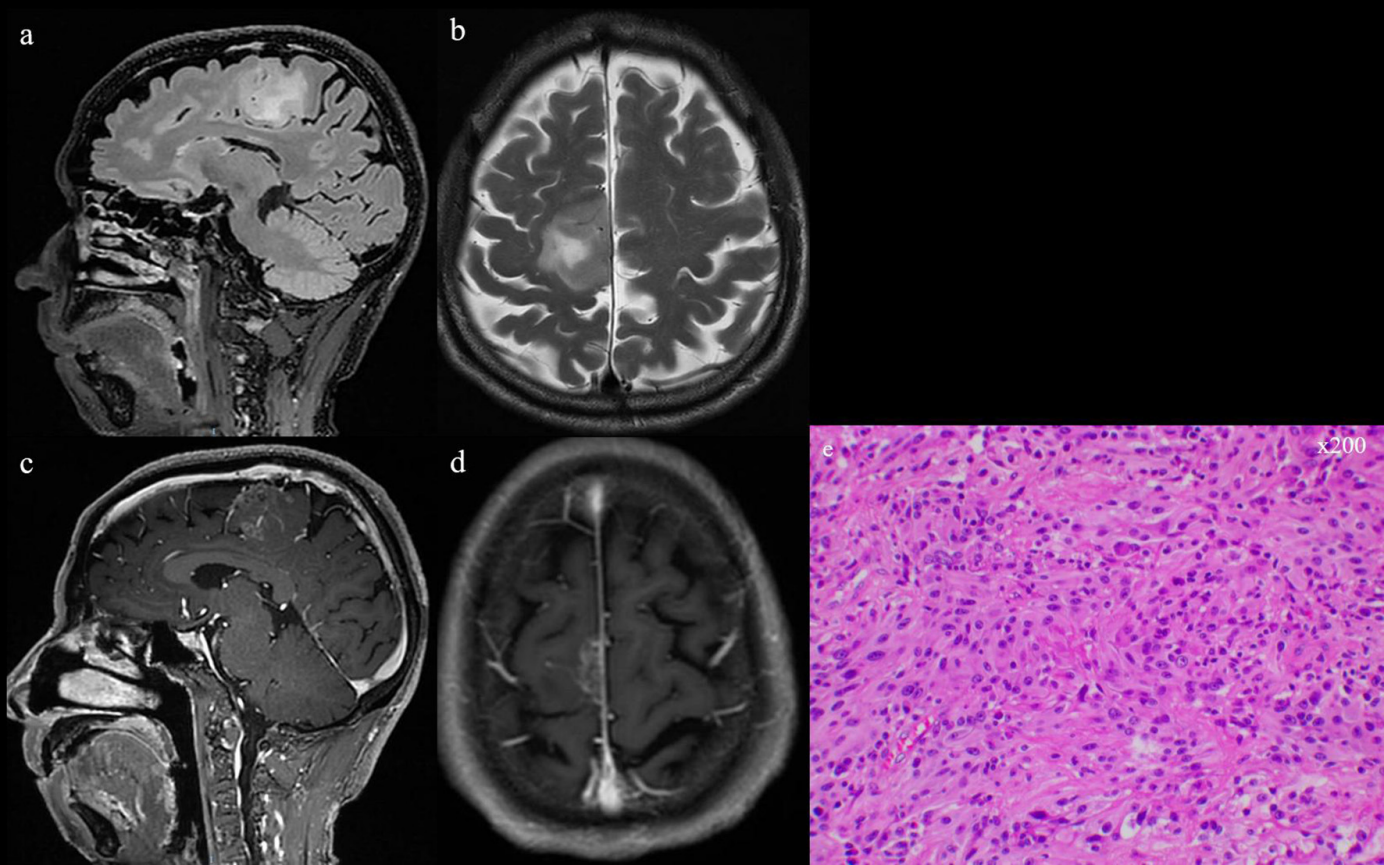
**(a, b)** T2 and flair series of preoperative MRI scans. **(c, d)** Preoperative MRI scan with gadolinium-based contrast agent, showing the occupying lesion in the left frontal lobe. **(e)** GBM confirmed by intraoperative pathologic examination (He, ×200).

**Figure 4:**
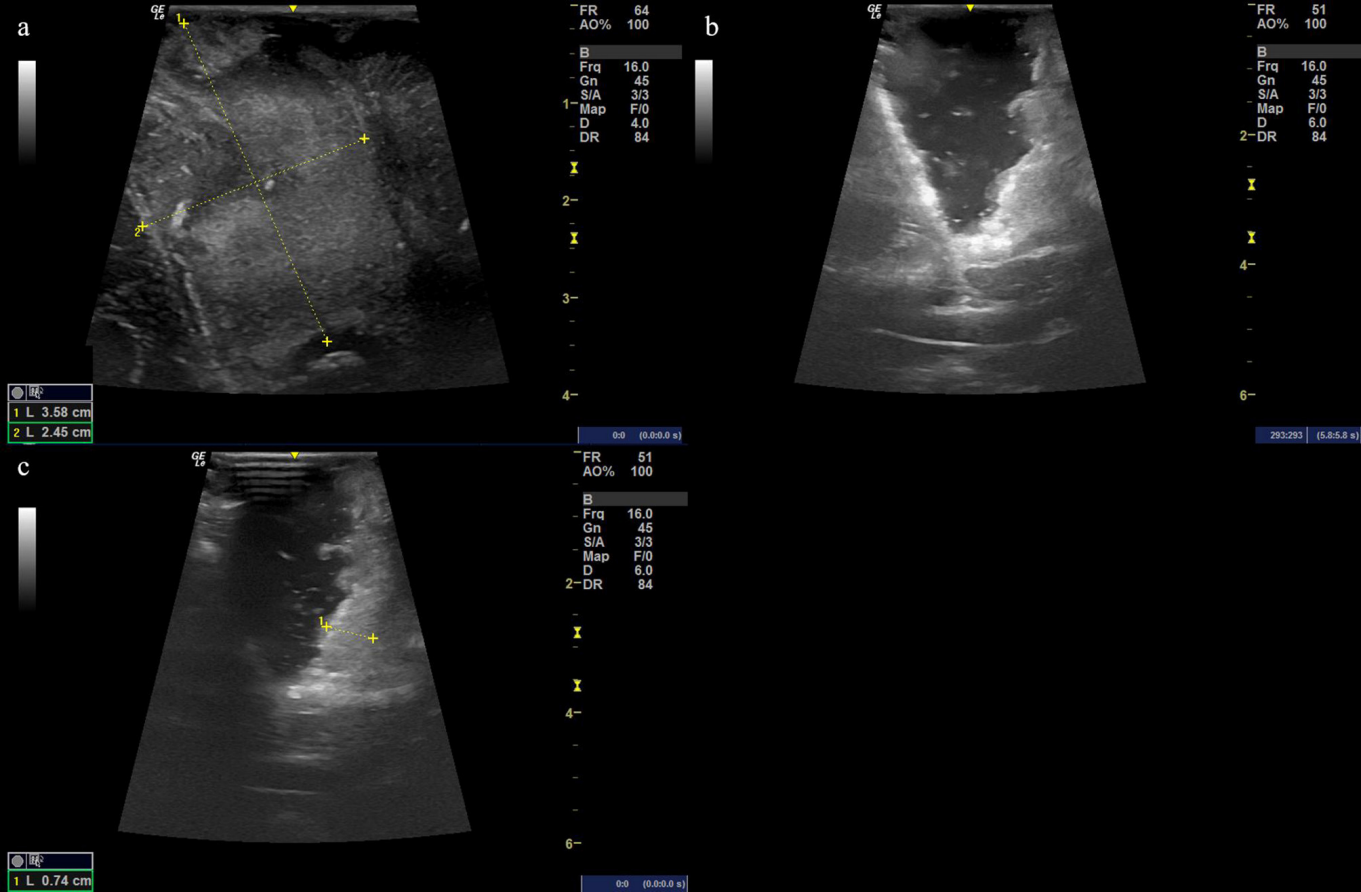
**(a)** Ultrasound image before cutting the dural matter displaying the occupying lesion of hyperechoic signal with the size of the possible solid tumor being 3.58 cm × 2.45 cm. **(b, c)** The resection procedure of a residual tumor guided by the intraoperative ultrasound scan, showing the extent of the resection area.

Given the economic factors discussed later in this context, the flexibility and economic benefits of linear array IOUS are sufficient to allow skilled neurosurgeons and sonographers to make an independent choice. IMRI can be used only in large medical centers with financial support as the cost of building an operating room with IMRI ranges from $3 to $7 million, and there are additional costs for staff training and equipment procurement. These costs may deter medical centers without sufficient financial support from adopting this technology (56). In comparison, IOUS ultrasound remains an inexpensive, real-time, safe, and reliable diagnostic technology that provides surgeons with real-time image guidance during surgery without radiation exposure. Additionally, as ultrasound probes and imaging technology continue to improve, IOUS is becoming increasingly widely used in high-grade glioma resection surgery. For example, some studies suggest that a high-frequency ultrasound probe can more accurately detect the normal brain tissue and vascular structures around the tumor, helping surgeons avoid damaging the surrounding tissue and vessels (57). Some clinical studies with a small number of cases also indirectly demonstrate the beneficial effect of IOUS on HGG resection (58). In summary, although different subtypes of high-grade glioma may not have distinct features on B-mode ultrasound, it remains a fast and effective diagnostic tool that provides real-time imaging information to guide surgery and evaluate surgical outcomes for doctors (Figures 3, 4).

Doppler ultrasound, on the other hand, can be used to guide surgical details. Steno et al. (59) demonstrated in low-grade glioma of the insula that Doppler ultrasound can display and avoid lenticulostriate arteries that supply descending motor pathways, resulting in all six cases included in the study, avoiding the risk of motor deficits and alleviating symptoms such as headaches and seizures. This suggests that during the growth of HGG, if important intracranial structures such as blood vessels and nerves are surrounded, Doppler ultrasound or even B-mode ultrasound can guide surgeons to avoid these critical structures, improve surgical outcomes, and ultimately improve patient prognosis. Given the complex vascular proliferation characteristics of HGG, ultrasound is expected to be a more effective tool for this purpose. Additionally, some studies have indicated that Doppler ultrasound not only detects blood vessels but also predicts brain displacement. Saß et al. (60) studied six patients with temporal lobe gliomas using 3D Doppler to visualize the vascular tree and compared the images with preoperative MRI at four points of interest. The purpose was to calculate the distance of brain displacement using 3D Doppler measurements, achieving the effect of measuring brain displacement (60). This leads to the shortcomings of IMRI, which, due to its long scanning time, can often be performed only once during surgery. However, changes in brain displacement occur in real time, and considering that HGG may grow in eloquent areas, the accuracy requirements for surgery are very high. Therefore, IMRI may not be able to provide a real-time update on brain displacement during surgery, which may impact surgical outcomes and patient prognosis. Other intraoperative imaging technologies, such as IOUS and ICT, may offer faster and more accurate alternatives for surgeons in these cases (6, 30, 61, 62). One way to overcome this is to use IOUS, which can scan and update images anytime and anywhere. Research has shown that real-time ultrasound can provide real-time excision progress images during long surgeries, promoting more complete excision (63–65).

Some investigators have already combined IOUS with 5-ALA and/or ICT to improve surgical outcomes (66, 67). Villa et al. recently investigated the feasibility of using fluorescence-guided surgery combined with IOUS for resection of HGG in five cases. They used fluorescein sodium (FS) and B-mode ultrasound + CEUS, fixed the ultrasound probe with electromagnetic tracking, and verified the results with postoperative enhanced MRI. Initially, they grossly removed the tumor using intraoperative yellow fluorescence and then used ultrasound guidance to clear any residual tumor. The combination of IOUS and fluorescein imaging demonstrated a higher surgical resection rate. Additionally, future studies should include more samples for investigation (68). This suggests a combined approach using imaging tools. A recent retrospective study by Hou et al. (41) included 40 patients who underwent IOUS and IMRI during glioma resection. IOUS was used for preoperative and intraoperative evaluations, while IMRI was used for final scanning to determine whether additional resection was necessary. The study results showed a total gross resection rate of 72.5%, which improved the quality control of resection and surgical outcomes compared to those of patients with assistance by IMRI alone. More combined methods were revealed in Barbagallo's research. In this study, 20 patients with recurrent GBM after radiochemotherapy underwent a second surgery with preoperative MRI and PET as well as intraoperative imaging using 5-ALA and IOUS to assist with resection. The multimodal surgical strategy showed that the combined use of 5-ALA and IOUS achieved a complete resection rate of 100% for all patients except for 2 despite slightly lower Sn and Sp (69).

There are also studies attempting to improve imaging using different types of coupling agents. Unsgård et al. (70) investigated a novel acoustic coupling fluid (ACF) by filling the surgical cavity with different concentrations of ACF and Ringer's solution in 15 patients with glioblastoma. The images collected were blindly evaluated by both the operating surgeons and specialized neurosurgeons. The results showed that ACF was more effective than Ringer's solution, and no adverse reactions were observed during the study period.

To improve methods for pathological examination, ultrasound can also be used. Patil et al. (71) compared the results of tumor biopsies using two different ultrasound techniques (89 cases by freehand 2D ultrasound and the rest by 3D navigational ultrasound) in 125 patients. The results showed that both freehand and 3D navigational ultrasound had high diagnostic rates, but the latter had slightly higher postoperative complications. The authors attributed this to the fact that cases requiring 3D navigational ultrasound were mostly deep-seated lesions. Di Somma et al. (72) combined neuroendoscopy and IOUS to perform biopsies on periventricular tumors. The results showed that the navigation was excellent, and the combined use of neuroendoscopy allowed for the timely detection of complications caused by biopsies, such as bleeding. In terms of the accuracy of ultrasound, studies suggest that navigation to the center of a solid mass is accurate, but sampling from the periphery may result in poor pathological detection rates (73).

CEUS has been highly anticipated in neurosurgery in recent years. Arlt et al. (74) studied its effectiveness in 50 brain tumor patients. Among 21 cases of GBM, 19 showed high uptake of the contrast agent, and 50% of the GBMs showed improved boundary imaging due to the use of the contrast agent (showing clearer edges). Similarly, approximately 50% of grade 2–3 tumors showed improved image quality. In 17 patients, five of them were detected with residual tumors during surgery and underwent further resection, with no deficits observed in any guided patient. CEUS can improve the display of HGG borders and increase resection effectiveness. This is similar to the study by Prada et al. (36). In a study including 120 cases of glioma, there were 76 cases of HGGs. Transmission electronic microscopic results were used to determine the Sn and Sp of CEUS-guided resection and similarly demonstrated relatively satisfactory Sn and Sp compared to CEUS (75). In a more recent study, Wu et al. (76) used CEUS to fuse preoperative MR images. They found that there were clear borders on CEUS in 13 out of 18 tumors with unclear boundaries on enhancement MRI (the “gold standard”) and that the CEUS images were correlated with the solid component area of enhancement MRI (73).

## Ultrasound application in pleomorphic/diffuse high-grade gliomas

There are few studies that discuss IOUS based solely on shape discrimination, with many studies classifying IOUS by the pathological type or whether surgery was performed after recurrence, such as the growth characteristics of GBM, which exhibit pleomorphic and diffuse growth, and a high recurrence rate after surgery. Considering that tumor reduction surgery is closely related to patient survival, ultrasound application is not affected by brain shift, can be used anytime and anywhere, and has no directional limitations, and technological advances have also changed its surgical approach. For example, a small-sized ultrasound probe (6–15 MHz) shaped like a hockey stick can penetrate deep into the surgical space for scanning in all directions. Even if the probe cannot penetrate deeply due to deep-seated tumors, scanning can still be performed on the surface of the brain using relatively low-frequency probes. Physiological saline can be injected into the surgical field to increase sound conduction, and tumor orientation, size, and other features can be observed by sacrificing some image quality (57). In some literature, occasional mention is made of the ultrasound effect on high-grade gliomas with indistinct borders. Although indistinct borders do not directly refer to diffuse growth characteristics, in the parts involved in such studies, although the surgical effect is not as good as that for low-grade tumors, traditional ultrasound imaging still proves to be effective and beneficial to patient survival and quality of life. Munkvold et al. (46) studied the IOUS-guided resection of diffuse gliomas (grades 2–4) and used postoperative MRI as the measure. The Sn of the last IOUS scan, which was the final measurement of the residual tumor, was 85%, while the Sp was 46%. Sp is better in low-grade gliomas but poorer in previously treated tumors. Considering the generally poor surgical outcomes of high-grade gliomas, such Sp should be considered satisfactory. The author's data analysis suggests that in this dataset, IOUS has poor detection for residual depth <1 mm, and the GTR rate is not related to image quality but only to tumor size and depth. According to the latest study by Wang et al. (77), which included 40 recurrent glioma patients who underwent IOUS-guided resection, compared with patients undergoing surgery without IOUS, B-mode ultrasound can detect glioblastoma multiforme in patients with multiple lesions that are difficult to distinguish on MRI, reduce recurrence rates, and increase the number of cases with complete non-recurrence, thus prolonging and increasing PFS and KPS. In conclusion, we believe that there can be further research on surgical outcomes specifically for irregularly shaped or diffusely growing tumors in this direction.

## Neurosurgery in pediatric patients with high-grade gliomas

The occurrence rate of HGGs in pediatric patients is 10% (78). Studies have shown that in pediatric neurosurgery, IOUS can rival IMRI in estimating the extent of resection and may have potential in HGGs (79–81). A new research study shows a good ability to localize the lesion accurately in all HGG cases (82). Generally, further research on pediatric HGGs with IOUS is needed to explore the surgical outcomes.

## Limitations of intraoperative ultrasound in current neurosurgery

IOUS technology has several advantages, including real-time imaging and not prolonging the operation time, but it does have some limitations that need to be addressed. The long and steep learning curve associated with the use of IOUS can be a challenge for some surgeons although training programs are available to help overcome this limitation. The use of IOUS is limited by bone, which can obstruct the transmission of sound waves. Despite these limitations, there is still room for further improvement in the imaging resolution and accuracy of lesion detection using IOUS technology. Advances in transducer design, image processing algorithms, and fusion imaging techniques are likely to further improve the precision and accuracy of IOUS imaging. Currently, more literature discusses the use of IOUS to detect brain shifts in real time, which itself has research potential. Intraoperative brain shift, mainly due to loss of cerebrospinal fluid in the patient, results in a “fatal” deviation from traditional preoperative MRI navigation. In the study by Steno et al. (59), surgeons performed semi-recumbent surgery and used bone wax to form a barrier to prevent cerebrospinal fluid loss. As a result, when examining patients during surgery, the impact of the brain shift was reduced. However, this method can also be applied to preserve fluids within the surgical cavity, thereby enhancing the ultrasound effect of IOUS (83).

A study that included 50 patients with IMRI and 17 patients with IOUS, with the metric being the Karnofsky Performance Status scale (KPS), compared the effect-cost ratio of IOUS vs. IMRI. The results showed that the IOUS group and the IMRI group had similar median scores on the scale, with slightly higher benefits for IMRI, but the difference between the two groups was not significant. Considering the actual benefits, the use of IOUS remains to be discussed, and its adoption depends more on economic factors (84).

## Future advancements in the application of artificial intelligence to IOUS

As ultrasound imaging technology continues to evolve, IOUS techniques will become more precise and efficient, with improved imaging quality providing more accurate lesion localization and surgical navigation to achieve better surgical outcomes. Multi-parametric ultrasound is also gradually being applied in the surgery of HGG (24, 28). The number of studies on the combination of IMRI, fluorescence imaging, ICT, and IOUS has increased gradually. In terms of artificial intelligence, researchers are attempting to use machine learning algorithms to analyze and classify IOUS images, with the aim of improving the accuracy and efficiency of ultrasound diagnosis. Nitsch et al. (85) utilized a novel MRI-US automatic segmentation and registration method, which outlined an image registration process using the falx cerebri and tentorium cerebelli as registration landmarks to improve registration accuracy and speed up the registration process. In future, real-time radiomics analysis technology holds great promise for improving the accuracy and efficiency of ultrasound imaging for brain tumor diagnosis and treatment. Radiomics refers to the extraction of quantitative imaging features from medical images, such as texture, shape, and intensity, which can be used for diagnostic and prognostic purposes.

The combination of radiomics with machine learning algorithms may make it possible to develop real-time image processing techniques that can automatically segment tumor borders and classify tumors based on their radiomic features. This could greatly improve the accuracy and efficiency of ultrasound imaging, allowing clinicians to make more informed decisions about patient care.

## Conclusion

IOUS technology shows great promise as an assistant tool for HGG operations and has the potential to improve surgical outcomes. However, similar to any technology, it does have some limitations that need to be addressed. With the continuous development of technology and the gradual application of artificial intelligence, IOUS technology is expected to become a more refined, efficient, and accurate assistant tool in future. Advances in real-time navigation, multiplane reconstruction, and fusion imaging techniques are likely to further improve the precision and accuracy of IOUS imaging. Moreover, the integration of artificial intelligence algorithms for automated image analysis and classification could reduce the subjectivity and variability associated with human interpretation of ultrasound images. Overall, IOUS technology holds great potential for improving the accuracy and efficiency of HGG surgery. Ongoing research and development in this area are likely to lead to further advancements in the field, enabling clinicians to provide better care for patients with brain tumors.

## Author contributions

RW drafted the initial manuscript. HC and JC reviewed and revised the manuscript. YC offered and described the pathology images. All authors reviewed and approved the final manuscript.
